# *SMN1* copy‐number and sequence variant analysis from next‐generation sequencing data

**DOI:** 10.1002/humu.24120

**Published:** 2020-10-14

**Authors:** Daniel Lopez‐Lopez, Carlos Loucera, Rosario Carmona, Virginia Aquino, Josefa Salgado, Sara Pasalodos, María Miranda, Ángel Alonso, Joaquín Dopazo

**Affiliations:** ^1^ Clinical Bioinformatics Area, Fundación Progreso y Salud (FPS) CDCA, Hospital Virgen del Rocio Sevilla Spain; ^2^ Computational Systems Medicine, Institute of Biomedicine of Seville (IBIS) Hospital Virgen del Rocio Sevilla Spain; ^3^ Genomic Medicine, Navarrabiomed Complejo Hospitalario de Navarra (CHN)‐Universidad Pública de Navarra (UPNA), IDISNA Pamplona Spain; ^4^ Bioinformatics in Rare Diseases (BiER), Centro de Investigación Biomédica en Red de Enfermedades Raras (CIBERER) FPS, Hospital Virgen del Rocío Sevilla Spain; ^5^ FPS/ELIXIR‐es, Hospital Virgen del Rocío Sevilla Spain

**Keywords:** next generation sequencing, pipeline, SMA

## Abstract

Spinal muscular atrophy (SMA) is a severe neuromuscular autosomal recessive disorder affecting 1/10,000 live births. Most SMA patients present homozygous deletion of *SMN1*, while the vast majority of SMA carriers present only a single *SMN1* copy. The sequence similarity between *SMN1* and *SMN2*, and the complexity of the SMN locus makes the estimation of the *SMN1* copy‐number by next‐generation sequencing (NGS) very difficult. Here, we present SMAca, the first python tool to detect SMA carriers and estimate the absolute SMN1 copy‐number using NGS data. Moreover, SMAca takes advantage of the knowledge of certain variants specific to *SMN1* duplication to also identify silent carriers. This tool has been validated with a cohort of 326 samples from the Navarra 1000 Genomes Project (NAGEN1000). SMAca was developed with a focus on execution speed and easy installation. This combination makes it especially suitable to be integrated into production NGS pipelines. Source code and documentation are available at https://www.github.com/babelomics/SMAca.

## 1 INTRODUCTION

Spinal muscular atrophy (SMA; MIM# 253300) is an autosomal recessive disorder caused by degeneration of alpha motor neurons in the anterior horn of the spinal cord, leading to hypotonia, muscular atrophy, and weakness of proximal muscles, predominantly affecting the lower extremities, being respiratory insufficiency a frequent cause of death in the most severe cases (Lunn & Wang, 2008). In most populations, SMA is caused by homozygous deletions of the survival motor neuron gene (*SMN1*). A total of 95%–98% of SMA patients show a complete absence of at least exon 7 of *SMN1*. Most of the remaining patients have a single copy of the *SMN1* gene which is inactive due to point mutations or deletion of exons 1–6. The disease severity is determined mainly by a copy gene, *SMN2*. The more *SMN2* copies present, the milder the phenotype usually is. Both genes, located on chromosome 5q13.2, can be distinguished by only five nucleotides (Monani et al., 1999).

Most individuals have two copies of each *SMN1* and *SMN2*, however, due to the complex genomic structure, gene conversion and rearrangements occur quite frequently in SMN locus leading to copy‐number variations (MacDonald et al., 2014). The majority of SMA patients have an *SMN1* deletion or gene conversion of *SMN1* into *SMN2*, which results in a homozygous loss of *SMN1* exon 7 or exons 7 and 8. Establishing the *SMN2* copy number is of importance for SMA patients due to the inverse correlation between disease severity and *SMN2* copy number. SMA carriers are mostly asymptomatic and can be identified by the presence of only a single *SMN1* exon 7 copy. About 5% of SMA carriers have two *SMN1* copies in *cis* in the same chromosome and 0 copies on the other (2 + 0) known as “silent carriers” (Alías et al., 2018). To detect silent carriers, we select samples with two polymorphisms (g.27134T>G [NC_000005.9:g.70247901T>G, rs143838139] and g.27706_27707delAT [NC_000005.9:g.70248473_70248474del, rs200800214]) associated with duplication events in *SMN1* (Luo et al., 2014). Depending on the number of *SMN2* copies, the expected should be close to 0.75 (2:1) or 0.5 (2:0) and, in both cases, the scaled coverage proportion of *SMN1* should be close to ½ in each position. Finally, a variant, referred to as *SMN1/2∆7‐8*, contains one or two extra copies of SMN exons 1–6 of *SMN1* or *SMN2*. This variant is often present in individuals with no, or only one, *SMN2*. Although it is frequently found (23%) in Spanish carriers and noncarriers, its clinical significance is not yet completely understood (Calucho et al., 2018). The frequency of SMA carriers in the population is around 1.7%–2.1% (Larson et al., 2015; Su et al., 2011), presenting most of them only a single *SMN1* exon 7 copy.

At present, the gold standard genetic test for SMA diagnosis is multiplex ligation‐dependent probe amplification (MLPA) of *SMN1* and *SMN2* although it cannot identify silent carriers (2 + 0) nor subtle mutations in *SMN1* (false‐negative rate of approximately 5%). However, next‐generation sequencing (NGS) technology is rapidly becoming a cost‐effective approach for clinical testing (Boycott et al., 2019). Despite the difficulties that the accurate determination of two genes almost identical inherent to short‐read technologies, some strategies to process NGS data have already been proposed to detect SMA carriers (Feng et al., 2017; Larson et al., 2015). Although at the moment of writing this manuscript there were not freely available tools to assess the mutational status of SMA from primary massive sequencing data, a tool was reported in a recent study published when this manuscript was under review (X. Chen et al., 2020).

Here, we present a python tool, SMAca that can detect SMA carriers and concomitantly estimate the absolute *SMN1* copy‐number from NGS data. Moreover, SMAca can exploit the knowledge variants specific to *SMN1* duplication to identify the silent carriers, following the recommendations for SMA carrier testing by the American College of Medical Genetics and Genomics (Prior et al., 2011).

## 2 MATERIALS AND METHODS

### 2.1 NGS data processing

The data were generated in the CNAG (Barcelona, Spain) with a NovaSeq 6000, using paired‐end reads (2 × 150 bp). Raw FASTQ files were processed following a standard NGS pipeline. Briefly, after filtering out low quality reads with *fastp* v0.20.0 (S. Chen et al., 2018), reads were aligned with *BWA–MEM* v0.7.16a (Li & Durbin, 2009) against the human reference genome GRCh37/h19. Potential polymerase chain reaction duplicates were marked with Picard v2.17.3 (http://broadinstitute.github.io/picard/). Finally, the whole set of BAM files were analyzed with SMAca in a single batch.

Additionally, a set of 1109 alignment CRAM files, comprising a diverse set of individuals from multiple populations, were downloaded from the 1000 genomes data portal (https://www.internationalgenome.org/data-portal/sample; 1000 Genomes Project Consortium, 2015; Clarke et al., 2017). The corresponding *SMN1* and *SMN2* validated copy number statuses were obtained from Vijzelaar et al. (2019). The whole set of CRAM files were analyzed with SMAca in a single batch. Samples with one experimentally validated SMN1 copy predicted to be SMA carriers were marked as true positives.

### 2.2 *SMN1* copy‐number estimation

The availability of a batch of samples allows a more accurate estimation of the *SMN1* copy number. SMAca first calculates the raw proportion of *SMN1* reads over the total number of reads covering *SMN1* and *SMN2* at three specific gene positions (denoted as *a*, *b*, and *c*) for each sample (Table 1).

**Table 1:** *SMN1* and SMN2 different nucleotides

Position	SMN1	SMN2
a	chr5:70247724	chr5:69372304
b	chr5:70247773	chr5:69372353
c	chr5:70247921	chr5:69372501

*Note*: Positions in *SMN1* (and the analogous positions in *SMN2*) used to calculate the raw proportion of *SMN1* reads(*D1ij*) over the total number of reads covering *SMN1* and *SMN2* (*D1ij *+* D2ij*).

These positions correspond to single nucleotide differences between *SMN1* and *SMN2*. Raw values are then scaled with respect to 20 control genes (Table 2) previously described to have consistent average coverage relative to *SMN1* and *SNM2* (Larson et al., 2015). Additionally, two genetic variants that have been associated with duplication events in *SMN1* are also screened and reported (Luo et al., 2014).

**Table 2:** Control genes

*ACAD9*	*FASTKD2*	*ITGA6*	*NTRK1*	*SIL1*
*ATR*	*FOXN1*	*IVD*	*PTEN*	*SLC22A5*
*CYP11B1*	*HEXB*	*LMNA*	*RAB3GAP1*	*SLC35D1*
*EDNRB*	*IQCB1*	*LRPPRC*	*RAPSN*	*STIM1*

*Note*: List of genes used to calculate the scale factor (${\hat{\theta }}_{i}$) and the scaled proportion of SMN1 reads (*π_ij_*).

In particular, the relative coverage of *SMN1* and *SMN2* with respect to each control gene is calculated: $Z_{\mathrm{ki}}=(c_{i1}+c_{i2})/H_{\mathrm{ki}}$, were $c_{i1}$ and $c_{i2}$ are the average coverage for the whole genes *SMN1* and *SMN2*, and $H_{\mathrm{ki}}$ is the average coverage for the control gene *k* in the *i*th sample. Then, the scale factor ${\hat{\theta }}_{i}=(\sum_{k=1}^{K}Z_{ki}/{\bar{Z}}_{k})/K$, where ${\bar{Z}}_{k}=(\sum_{i=1}^{N}Z_{ki})/N$, *N* is the total number of samples and *K* the total number of control genes, is calculated for each sample. Finally, the raw proportion of *SMN1* reads are scaled: $\pi_{\mathrm{ij}}={\hat{\theta }}_{i}\times D_{1\mathrm{ij}}/(D_{1\mathrm{ij}}+D_{2\mathrm{ij}})$, where $D_{1\mathrm{ij}}$ and $D_{2\mathrm{ij}}$ are the raw coverage for *SMN1* and *SMN2* at position j in the *i*th sample.

### 2.3 SMA carrier categorization

Results were classified following some simple rules. Samples with a scaled coverage proportion of *SMN1* ($\pi_{\mathrm{ij}}$) less than ⅓ in positions *a*, *b*, or *c* were marked as likely carriers. The scale factor ${\hat{\theta }}_{i}$ (i.e., proportional to the total *SMN1* and *SMN2* copy number) and the raw proportion of *SMN1*/*SMN2* depth of coverage at positions *a*, *b*, and *c* ($D_{1\mathrm{ij}}/D_{2\mathrm{ij}}$), were used to estimate the absolute copy‐number as follows:

- Genotypes 1 *SMN1*:3 *SMN2* are expected to have ${\hat{\theta }}_{i}$ ∼ 1 and $D_{1\mathrm{ij}}/D_{2\mathrm{ij}}$ ∼ ⅓.
- Genotypes 1 *SMN1*:2 *SMN2* are expected to have ${\hat{\theta }}_{i}$ ∼ 0.75 and $D_{1\mathrm{ij}}/D_{2\mathrm{ij}}$ ∼ ½.
- And genotypes 1 *SMN1*:1 *SMN2* are expected to have ${\hat{\theta }}_{i}$ ∼ 0.5 and $D_{1\mathrm{ij}}/D_{2\mathrm{ij}}$ ∼ 1.

To detect silent carriers, we select samples with two polymorphisms (g.27134T>G and g.27706_27707delAT) associated with duplication events in *SMN1* (Luo et al., 2014). Depending on the number of *SMN2* copies, the expected ${\hat{\theta }}_{i}$ should be close to 0.75 (2:1) or 0.5 (2:0) and, in both cases, the scaled coverage proportion of *SMN1* should be close to ½ in each position.

Therefore, SMA carriers can be detected either in genome or exome sequences, and even small panels as long as SMN locus and control genes are covered. However, for silent carriers, which require the analysis of some intronic positions, the use of genomic sequences is recommended.

## 3 RESULTS

### 3.1 Experimental validation

To test the reliability of SMAca predictions in a real scenario, we leveraged our participation in the Navarra 1000 Genomes Project NAGEN1000 to screen a dataset of 326 genomes. Among them, seven samples (2.15%) were identified as putative SMA carriers and successfully validated by MLPA (Table S1a–d). Interestingly, the percentage of the predicted and further confirmed SMA carriers in our dataset fits perfectly to the expected carrier frequency (2.10%) previously described in the bibliography (Su et al., 2011). Moreover, the genotype estimation for the SMA carrier samples agreed with the experimental validation as shown in Table 3 (except for case no. 7 where the genotype could not be estimated). Interestingly, case no. 1 corresponds to an SMA carrier with an extra copy of SMN exons 1–6 (*SMN1/2∆7‐8*).

**Table 3:** MLPA results

No. of id	PI_a	PI_b	PI_c	cov SMN1a	Cov SMN1b_e7	Cov SMN1c	Cov SMN2a	Cov SMN2b e7	Cov SMN2c	Scale factor	CN estimation	MLPA genotype
1	0.26	0.19	0.24	13	8	11	23	23	22	0.741	1:2	1:1*
2	0.29	0.22	0.29	17	14	21	46	54	57	1.101	1:3	1:3
3	0.22	0.22	0.21	15	15	13	42	41	38	0.842	1:2	1:2
4	0.32	0.26	0.23	27	18	13	42	39	33	0.837	1:2	1:2
5	0.28	0.26	0.22	25	24	20	72	78	79	1.109	1:3	1:3
6	0.30	0.26	0.25	23	22	19	65	74	68	1.148	1:3	1:3
7	0.40	0.32	0.30	23	19	18	30	36	37	0.936	Inconclusive	1:2

*Note*: PI_x: scaled proportion of SMN1 reads in position x; cov xp: raw coverage of gene x at position p; scale factor: ${\hat{\theta }}_{i}$; CN estimation: absolute copy number estimation SMN1:SMN2; MLPA genotype: genotype inferred from MLPA analysis.

Abbreviation: MLPA, multiplex ligation‐dependent probe amplification.

* MLPA analysis showed deletion of exons 7–8 on both genes but three copies of exons 1–6 (impossible to distinguish whether they come from SMN1 or SMN2).

Additionally, we have performed a large‐scale validation of 1109 genomes from a diverse set of individuals from multiple populations. Results show an overall high accuracy (0.998) and an F1 score (Jackson et al., 1989) of 0.938 (see Table S1 with the validation test, Table S2 with the full SMAca output, and Table S3 with the list of validated samples).

### 3.2 Performance

**Figure 1:**
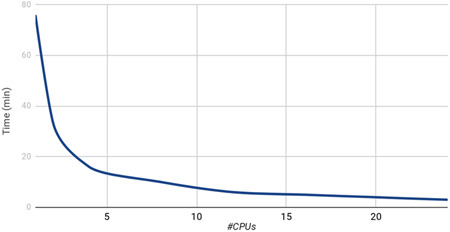
SMAca performance. Elapsed time for the analysis of the dataset (326 whole genome sequences) and different numbers of CPUs. The analysis of the whole dataset takes only 3 min by using 24 threads

With the idea of facilitating the introduction of SMAca in production NGS pipelines, it has been optimized for running in different computer environments. Special stress has been made in the parallelization for exploiting multiple cores/processors when available. Figure 1 shows the runtimes with an increasing number of processors. The estimation of SMA mutational and copy number status for 326 genomes from Navarra 1000 Genomes Project NAGEN1000 takes almost 1 h and a half in one core but can be reduced to only 3 min in 24 cores (see Figure 1). When a similar number of samples is analyzed with the recently published tool (X. Chen et al., 2020) in the same conditions, the runtime exceeds 1.5 days.

## 4 CONCLUSIONS

Here, we present SMAca, the first freely available python tool to detect SMA carriers and estimate the absolute *SMN1* copy‐number from NGS data. As a conceptual novelty, SMAca includes the analysis of two polymorphisms that have been linked to silent carriers (Luo et al., 2014) and are recommended for SMA carrier testing by the American College of Medical Genetics and Genomics (Prior et al., 2011). This tool was developed with a focus on execution speed and easy installation. Thus, SMAca is available through Bioconda (Grüning et al., 2018) to facilitate distribution and foster reproducibility (Baker, 2016). Also, SMAca is robust against technical biases, including read length, sequencing platform, capture method, or the aligner used. Moreover, given the way in which SMAca carries out the data normalization, using the depth of coverage of *SMN1* + *SMN2*, the results must be consistent across different populations given that the total SMN copy number tends to be constant (Vijzelaar et al., 2019). This combination makes of SMAca an especially attractive tool to be integrated into production NGS pipelines.

## CONFLICT OF INTERESTS

The authors declare that there are no conflict of interests.

## Supporting information

Supporting information.

Supporting information.

## Data Availability

The data that support the findings of this study are available from Navarra 1000 Genomes project (NAGEN1000). Restrictions apply to the availability of these data, which were used under license for this study. Another dataset of validation composed of 1109 alignment CRAM files is available at https://www.internationalgenome.org/data-portal/sample. SMAca source code and documentation are available at https://www.github.com/babelomics/SMAca.
