# Supplementary material for: SMN1 copy‐number and sequence variant analysis from next‐generation sequencing data
Source: Hum Mutat. 2020 Oct 14;41(12):2073–7. doi: 10.1002/humu.24120 (PMC7756735; doi:10.1002/humu.24120)
Supplement: Supplementary file 1 — Supporting information. [file HUMU-41-2073-s001.pdf]

Supplementary Figure S1. **Multiplex Ligation-dependent Probe Amplification (MLPA) analysis.** The SALSA MLPA probemix P021-B1 SMA-v02 kit is a multiplex PCR technique that uses a single primer pair to amplify up to 32 probes, each with a unique genomic target and length between 175 and 445nt. Four probes are specific for sequences in exon 7 or 8 of either *SMN1* or *SMN2*. 17 probes detect sequences present in both *SMN1* and *SMN2*. There is one probe for the *NAIP* gene and ten reference probes. PCR amplicons are fluorescently labelled and separated and quantified by capillary electrophoresis (Applied Biosystems 3500 Genetic Analyzer) following the MLPA® General Protocol (MRC-Holland). By comparing the resulting peak pattern of a sample to those of a set of reference samples, the number of genomic targets present in the sample of interest can be determined (Coffalyser MLPA analysis software; MRC-Holland). Coffalyser displays lower border (red) and upper border (blue) in ratio charts. In general, when a probe ratio crosses these borders, it is indicative for a duplication or deletion, assuming that the normal copy number of the sequence targeted by the probe is two (Coffalyser Reference Manual, MRC-Holland).

MLPA results for samples identified as putative SMA carriers

#4

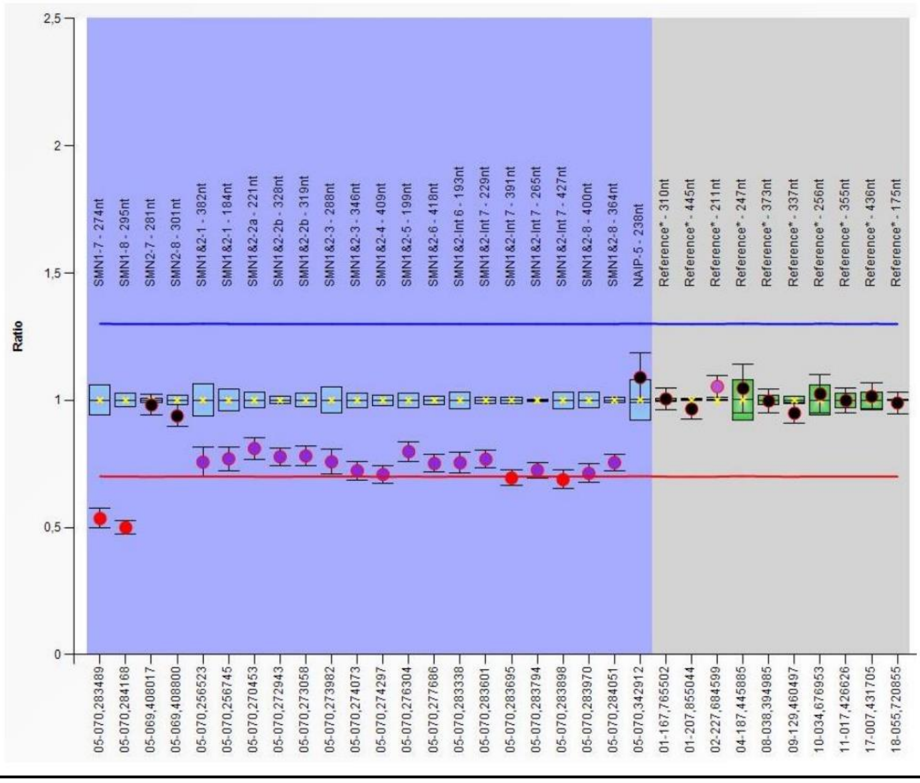

Figure S1A: SMA carrier with a single *SMN1* copy and two copies of *SMN2* (cases #7 and #4)

#6

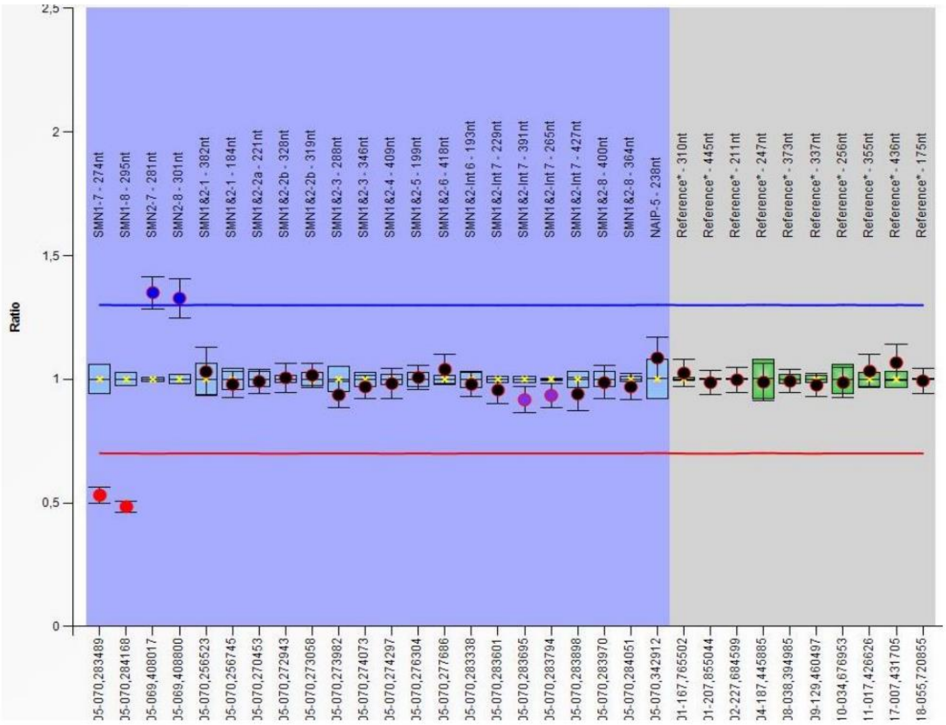

Figure S1B: SMA carrier with a single *SMN1* copy and three copies of *SMN2* (cases #2, #5 and #6)

id	MLPA result
#1	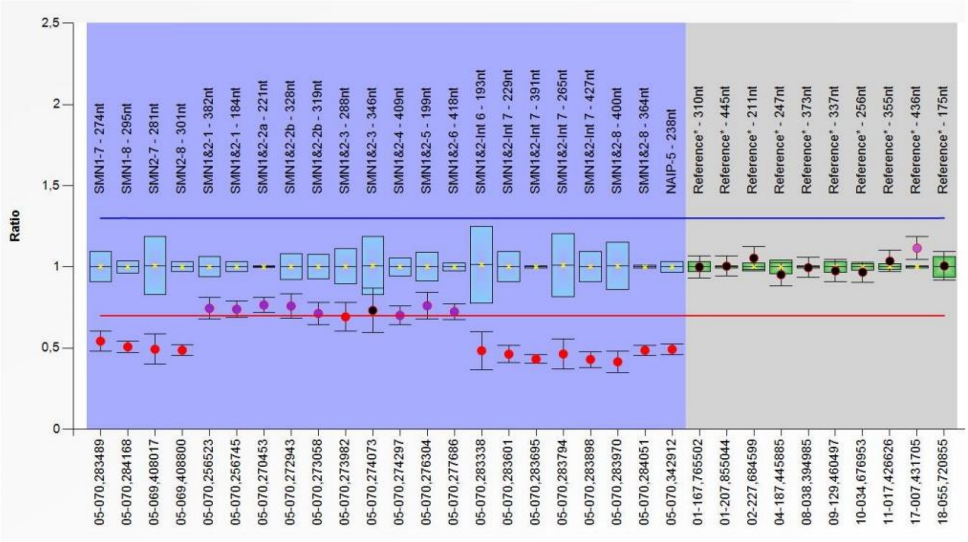

Figure S1C: SMA carrier with a single *SMN1* copy, a single copy of *SMN2* and an extra copy of exons 1-6 of *SMN1* or *SMN2* (*SMN1/2Δ7-8)* (case #1)

#3

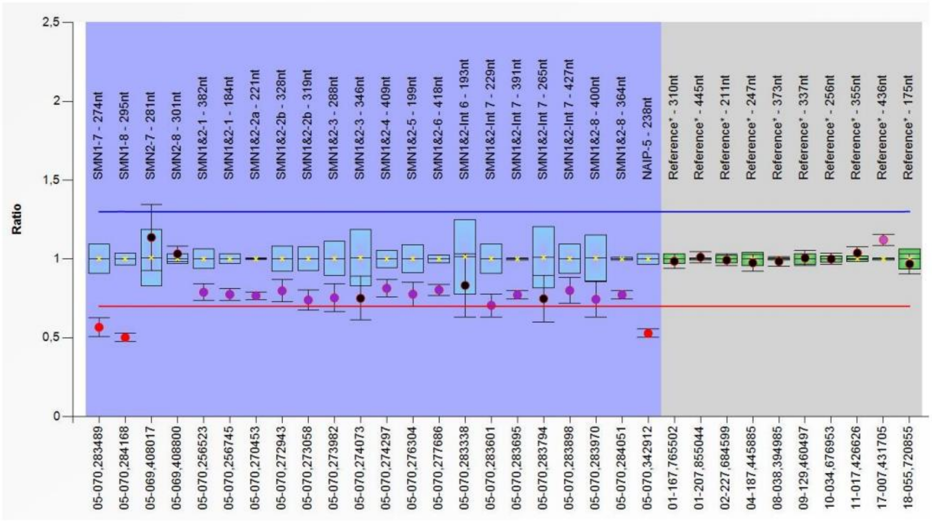

Figure S1D: SMA carrier with a single *SMN1* copy, two copies of *SMN2* and one copy of NAIP gene (exon 5) (case #3). Some publications indicate that patients with fewer copies of NAIP have more severe phenotypes than patients with more copies.
